# Antiplatelet therapy does not influence outcome or host response biomarkers during sepsis: a propensity-matched analysis

**DOI:** 10.1186/cc14110

**Published:** 2015-03-16

**Authors:** MA Wiewel, SF De Stoppelaar, LA Van Vught, JF Frencken, AJ Hoogendijk, PM Klein Klouwenberg, J Horn, MJ Bonten, MJ Schultz, AH Zwinderman, OL Cremer, T Van der Poll

**Affiliations:** 1 Academic Medical Center, University of Amsterdam, the Netherlands; 2 University Medical Center Utrecht, the Netherlands

## Introduction

Sepsis is a life-threatening condition, during which triggering of inflammatory and coagulation cascades, together with endothelial damage, invariably leads to activation of platelets. Although platelets are essential components of primary hemostasis, uncontrolled platelet activation during sepsis may contribute to organ failure. The aim of this study was to investigate whether chronic antiplatelet therapy impacts on the presentation and outcome of, and the host response to, sepsis.

## Methods

We performed a prospective observational study in patients admitted with sepsis to the mixed ICUs of two hospitals in the Netherlands between January 2011 and July 2013. Cox proportional hazards regression was used to estimate the effect of antiplatelet therapy on mortality. To account for indication bias, a propensity score was constructed, and used to match antiplatelet therapy users to nonusers. Plasma biomarker levels, providing insight into hallmark host responses to sepsis, including activation of endothelial cells and the cytokine network, were determined during the first 4 days after ICU admission.

## Results

Of 1,070 sepsis patients, 297 (27.8%) were on antiplatelet therapy, including acetylsalicylic acid, clopidogrel and dipyridamole, prior to ICU admission. Antiplatelet users and nonusers differed significantly with regard to several baseline characteristics, such as age, gender and cardiovascular disease. Antiplatelet therapy was not related to sepsis severity at presentation, the primary source of infection, causative pathogens, the development of organ failure or shock during ICU stay, or mortality up to 90 days after admission, in either the unmatched or propensity-matched analyses. Antiplatelet therapy did also not modify plasma concentrations of biomarkers.

## Conclusion

Pre-existing antiplatelet therapy does not influence clinical disease severity at presentation, nor the host response or outcome following sepsis.

## Acknowledgements

This research was performed within the framework of CTMM, the Center for Translational Molecular Medicine (http://www.ctmm.nl), project MARS (grant 04I-201).

